# Disability-adjusted life years associated with COVID-19 in Brazil, 2020

**DOI:** 10.1371/journal.pone.0319941

**Published:** 2025-03-27

**Authors:** Cleber Vinicius Brito dos Santos, Lara Esteves Coelho, Guilherme Tegoni Goedert, Paula Mendes Luz, Guilherme Loureiro Werneck, Daniel Antunes Maciel Villela, Cláudio José Struchiner

**Affiliations:** 1 Departamento de Epidemiologia, Instituto de Medicina Social, Universidade do Estado do Rio de Janeiro (UERJ), Rio de Janeiro, Brazil; 2 Instituto Nacional de Infectologia Evandro Chagas, Fundação Oswaldo Cruz (FIOCRUZ), Rio de Janeiro, Brazil; 3 Escola de Matemática Aplicada, Fundação Getúlio Vargas (FGV), Rio de Janeiro, Brazil; 4 Instituto de Estudos em Saúde Coletiva, Universidade Federal do Rio de Janeiro (UFRJ), Rio de Janeiro, Brazil; 5 Programa de Computação Científica, Fundação Oswaldo Cruz (FIOCRUZ), Rio de Janeiro, Brazil; Facultad Latinoamericana de Ciencias Sociales Mexico, MEXICO

## Abstract

**Background:**

We quantified the national- and state-level burden of COVID-19 in Brazil and its states during 2020 and contrasted it to the burden from other causes of disease and injury.

**Methods:**

We used national surveillance data on COVID-19 cases, hospitalisations and deaths between February/2020 to December/2020. We calculated disability-adjusted life years (DALYs) based on the COVID-19 consensus model and methods developed by the European Burden of Disease Network, which includes mild to moderate, severe, and critical COVID-19 cases, long covid and deaths due to COVID-19. We used Brazil DALYs estimates from the Global Burden of Disease Collaborative Network to compare the COVID-19 burden to that from other causes of disease and injury.

**Results:**

COVID-19’s led to 5,445,785 DALYs, or 2,603 DALYs/100,000, with > 99% of the burden caused by mortality. Males accounted for the largest fraction of DALYs (3,214,905 or 59%) and DALYs per 100,000 population (140,594 or 63%). Most populated states experienced the highest DALYs. However, the DALYs per 100,000 population were higher in the states of Rio de Janeiro (4,504 DALYs/100,000), Amapá (4,106 DALYs/100,000) and Roraima (3,981 DALYs/100,000). Assuming no major changes in disease burden from other causes of disease and injury from 2019 to 2020 in Brazil, COVID-19’s burden would rank as the leading cause of disability in 2020.

**Conclusions:**

Compared with studies with similar methodology, our findings showed that Brazil experienced the highest COVID-19 burden (per 100,000 population) in the world. COVID-19 severely impacted Brazil’s populational health in 2020, highlighting the lack of effective mitigation efforts.

## Background

In December 2019, the first outbreak caused by the severe respiratory syndrome coronavirus 2 (SARS-CoV-2) was identified in Wuhan, China. Later in March 2020, the World Health Organization (WHO) declared the Coronavirus disease 2019 (COVID-19) a pandemic [1]. In Brazil, the first case was reported on February 26, 2020. Over that year, 7 million cases and 190,000 deaths were registered, and the inability of Brazil’s federal government to develop a nationwide plan to combat the pandemic directly affected the implementation of public health measures to control the spread of the disease [2–4].

Since the start of the pandemic, many efforts have been made to measure the health impact of COVID-19 on the population, including monitoring and daily publication of new cases, hospitalisation and deaths[2]. In Brazil, the Ministry of Health is responsible for COVID-19 surveillance, including monitoring and reporting case counts, hospital admissions, and deaths. Additionally, a myriad of studies have been undertaken focusing on different perspectives of the disease, including national and local seroprevalence surveys [5,6], estimates of excess mortality[7], and evaluations of the impact of vaccination, social vulnerability and mobility on COVID-19 incidence and mortality[8–11]. All these studies have helped to monitor the evolution of the pandemic over space and time and quantify the effects of measures to reduce disease incidence.

Nevertheless, an overall assessment of the health burden of COVID-19, which accounts for the disease’s morbidity and mortality in a single metric, can be of great use in facilitating the comparison with other countries and diseases. This can be achieved by standardising the population health loss due to both cases and deaths as a function of time, using the disability-adjusted life years (DALYs) metric [12–14]. The morbidity is translated into estimates of years lived with disability (YLD), adjusting for the severity of the disability caused by the disease or injury. The mortality is translated into years of life lost due to premature mortality (YLL), using age-conditional life tables, considering that deaths at younger ages have a more significant impact on population health[12–14].

This study aimed to estimate the direct impact of COVID-19, measured in DALYs, on the Brazilian population’s health during 2020 and to contrast COVID-19’s burden with that from other causes of disease and injury.

## Methods

### Data

We used data from four national databases: (i) Flu-like syndrome (*Síndrome Gripal*) from the E-SUS Notifica, which includes anonymised individual-level data on suspected cases of COVID-19 [15,16]; (ii) Severe Acute Respiratory Infection/Illness (SARI) from the Influenza Epidemiological Surveillance System (SIVEP-Gripe) [17], which holds anonymised individual-level data on all COVID-19 severe cases that led to hospitalisation; (iii) the Mortality Information System (*Sistema de Informações sobre Mortalidade - SIM*)[18] database, which includes anonymised individual-level data on all deaths registered in the country, and (iv) Brazilian Institute for Geography and Statistics (IBGE) database on sex and age-specific population estimates at the national and state levels and shapefiles of the Brazilian territory[19–21].

We filtered the disease and death records which contain information on sex, age, state and symptom onset/hospitalisation onset between February 26, 2020, and December 31, 2020. The COVID-19 deaths were those where the primary cause of death was coded using the WHO International Classification of Disease 10^th^ revision (ICD-10) codes U071 (COVID-19, Virus identified), U072 (COVID-19, Virus not identified), B342 (Coronavirus infection unspecified), B972 (Coronavirus as the cause of diseases classified elsewhere), U109 (Multisystem inflammatory syndrome associated with COVID-19, unspecified)[22]. To estimate the YLD, the disease registries were aggregated by five-year age group, sex, and state. The death registries followed the same grouping as the disease registries, except that the under-5-year age group was split into under-one-year old and 1-4 years of age. The oldest age group was set at 95 years or older.

To compare the COVID-19 burden to the burden from other causes of disease and injury in Brazil, we used data from the GBD results tool from the Global Burden of Disease Collaborative Network of the Institute for Health Metrics and Evaluation (IHME)[13,23]. Burden of disease estimates for 2020 was not available by the time of the preparation of the manuscript; hence we used 2019 DALY estimates for both sexes, including all ages and grouped the estimates by level-3 causes. The estimates are freely available on the IHME website (https://vizhub.healthdata.org/gbd-results/), and the resulting dataset is presented in S1 Table.

### Disability-Adjusted Life Year (DALY)

The DALY is a health metric measuring the healthy life years lost due to a disease. DALYs are estimated by summing the number of years of life lost due to premature mortality (i.e., YLLs) and the number of years lived with disability, adjusted for the severity of the disease (i.e., YLDs) [12]. When estimating DALYs from COVID-19, we accounted for all health states experienced upon infection and development of symptoms, which were classified as “mild to moderate”, “severe”, “critical”, “long COVID”, and death due to COVID-19.

### Years of life lost due to premature mortality (YLL)

YLLs were calculated by multiplying the number of deaths in each age group and sex by the residual life expectancy at the age of death:


YLL=DeathsXRLE

where *RLE* corresponds to the Residual Life Expectancy. We used the age-conditional life expectancy defined by the GBD 2019 reference life table [24].

### Years lived with disability (YLD)

We rely our analysis in methods previously defined by Wyper et al [25] and used to estimate the burden of COVID-19 in other studies (see for example [26,28]). To estimate the severity of each health state, we obtained disability weights (DWs) from the 2019 Global Burden of Disease study (2019 GBD study) and the European Disability Weight (EDWS) study [25–29]. The DW reflects the severity of a health state (i.e., the reduction in the quality of life). For each health state, there are three parameters as inputs: number of cases from the surveillance systems, duration of the health state and the disability caused by the health state. The YLD was calculated by summing the product of these three parameters as follows:


YLD=NumberofcasesXDurationXDW

We defined mild to moderate cases as all confirmed COVID-19 diagnoses that did not result in hospitalization (i.e., we filtered the confirmed cases and removed the hospitalized cases in each age group, sex and state strata). Severe cases were defined as hospitalised cases that did not require intensive care. We classified “critical cases” as those requiring intensive care. To estimate the YLD due to “long COVID”, we followed previous studies and assumed that approximately 1-in-7 patients (i.e., 13.3%) of mild to moderate cases would suffer post-acute consequences for 28 days, reflecting evidence from the literature [14,25,30,31]. Given that the E-SUS database does not provide the duration of mild to moderate cases, we defined the mean duration of this health state as ten days as proposed by the Center For Disease Control and Prevention (CDC) and also applied in similar studies (see for example [15,31–33]). We calculated the duration of “severe” cases as the mean duration of hospitalisations not requiring ICU admission and of “critical” cases as the mean duration of hospitalizations requiring ICU admission. Duration of hospitalization was calculated from hospital admission and discharge dates which are available in the SIVEP-Gripe database [17]. The names, descriptions and disability weights of the health states “mild to moderate”, “post-acute consequences” (i.e., long covid) and “severe” were based on those from the GBD 2019 study for infectious diseases of the lower respiratory tract [14]. Lastly, the health state “critical” was defined by the European Disability Weight study [34].

The health states definitions, DW from the GBD and EDWS and the data sources are shown in Table 1. To explore spatial differences in the COVID-19 burden across the country, in a secondary analysis we estimated the DALYs for each Brazilian state in 2020.

**Table 1:** COVID-19 health states, attributable disability weights and data sources.

Health state	Health state description	Data	Duration (days)	Data source	Disability weight (95% uncertainty interval)
Mild to moderate	Non-hospitalised symptomatic case, which causes some difficulty with daily activities	Number of symptomatic cases (non-hospitalised)	10[33]	E-SUS[16]	0.051 (0.032-0.074)
Severe	Hospitalised symptomatic case, which causes great difficulty with daily activities	Number of hospitalised patients (non-intensive care)	Estimated mean duration of hospital stay	SIVEP-Gripe[17]	0.133 (0.088-0.190)
Critical	Hospitalised symptomatic case requiring intensive care unit admission with or without respiratory support.	Number of hospitalised patients (intensive care)	Estimated mean duration of ICU stay	SIVEP-Gripe[17]	0.655 (0.579-0.727)
Long COVID (Post-acute consequences)	Suffers from a symptomatic health loss post-infection, such as fatigue, emotional lability, insomnia etc.	Estimated based on the fraction (13.3%) of mild to moderate cases that evolved to long covid (1-in-7) [25]	28[30]	E-SUS[16]	0.219 (0.148-0.308)

ICU: Intensive Care Unit

### Uncertainty and sensitivity analysis

Following similar studies, we used Monte Carlo simulations from a beta PERT distribution to sample random values from the uncertainty distribution of the DW range values [31,35–37]. In each of the 10,000 iterations, the DW sample values were used to calculate a YLD estimate. The combination of iterations results in an empirical distribution of YLD estimates, reflecting the joint uncertainty in the input parameters (i.e., the DW range values), which were summarised by its 95% bootstrapped uncertainty intervals (UI) and will be presented in Table 3.

Furthermore, we used a univariate sensitivity analysis to quantify the impact of the uncertainties around the number of COVID-19 mild to moderate, severe and critical cases and the proportion of cases that suffer from long COVID [31]. This sensitivity analysis was based on assuming two scenarios: a lower-bound scenario, where we decrease the duration and the number of cases in each health state by half, and an upper-bound scenario, where we double the health state duration and the number of cases, respectively (presented in S2 Table).

All analyses were run in R version 4.1.2; and the R code for analyses is available in S1 Text [38].

## Results

From February 26, 2020, to December 31, 2020, more than 7.8 million mild to moderate, severe, and critical COVID-19 cases and 221,012 deaths were notified in Brazil (Table 2). Based on the number of mild to moderate cases, we estimated that 942,263 persons suffered from long covid, such as fatigue and insomnia (Table 2).

**Table 2:** Absolute number of observed COVID-19 mild to moderate, severe, and critical cases, and of deaths due to COVID-19, and estimated number of long covid cases in Brazil, by age group and sex, between February 26, 2020, and December 31, 2020.

Age group	Mild cases		Long covid		Severe cases		Critical cases		Deaths	
	Female	Male	Female	Male	Female	Male	Female	Male	Female	Male
0-4 years	48571	59751	6460	7947	9973	12957	1900	2340	237	275
5-9 years	48951	65774	6510	8748	6252	8158	1007	1251	50	65
10-14 years	63810	74438	8487	9900	3369	3800	564	681	62	76
15-19 years	134577	121308	17899	16134	3806	2629	589	498	194	201
20-24 years	321839	301734	42805	40131	6938	4900	979	889	367	411
25-29 years	416798	382358	55434	50854	9014	7974	1375	1587	525	706
30-34 years	446916	415186	59440	55220	11523	12689	1855	2623	926	1431
35-39 years	473536	431249	62980	57356	14306	18163	2650	4025	1578	2435
40-44 years	444606	393178	59133	52293	15251	21948	3008	4985	2278	3690
45-49 years	353852	305690	47062	40657	16172	23905	3468	5951	3061	5072
50-54 years	293577	249889	39046	33235	18957	26327	4395	7536	4301	7087
55-59 years	239565	205887	31862	27383	21821	28815	5767	8996	6212	10012
60-64 years	164777	147879	21915	19668	23509	29061	7066	10463	8768	13389
65-69 years	108902	100710	14484	13394	23708	29287	7895	11312	10769	16515
70-74 years	66427	63031	8835	8383	22993	27033	8144	10714	11943	17524
75-79 years	37415	34914	4976	4644	20851	22187	7511	9285	12162	16210
80-84 years	18740	18350	2492	2441	19802	18672	7049	7280	12422	14445
85-89 years	9461	9415	1258	1252	15239	12129	5224	4577	10032	9821
90 or + years	5569	6056	741	805	12892	7969	3689	2520	8988	6772
**Total**	3697889	3386797	491819	450444	276376	318603	74135	97513	94875	126137

**Fig 1:**
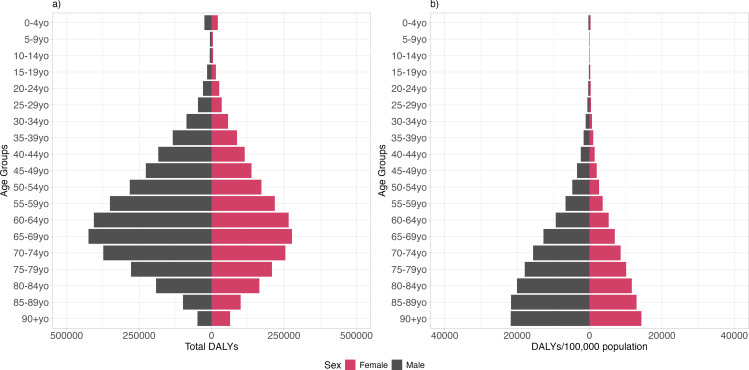
Distribution of estimated (A) Disability-adjusted life years (DALY) and (B) DALY per 100,000 individuals due to COVID-19 in Brazil by age group and sex between February 26, 2020, and December 31, 2020.

The estimated COVID-19 burden in 2020 was 5,445,785 DALYs (95% uncertainty interval (UI) 5,438,752-5,458,732), corresponding to 2,603 DALYs per 100,000 population. For both sexes, more than 99% of the total DALY burden was experienced by individuals between 30 and 84 years of age, with the higher burden experienced by males between 55- and 74-year-old groups (Fig 1a and Table 3). The DALY rate showed that, for both sexes, most of the burden was experienced by individuals aged 60 years or more with an in increase on the number of DALYS with ageing (Fig 1b and Table 3). Irrespective of the age group, males lost more DALYs due to COVID-19 than females (Fig 1b and Table 3).

**Table 3:** Total number of years of life lost due to disability (YLD), years of life lost due to premature mortality (YLL) and Disability Adjusted Life Years (DALYs) due to COVID-19 in Brazil by age group and sex between February 26, 2020, to December 31, 2020.

Age group	YLD*				YLL				DALYs*			
	Female		Male		Female		Male		Female		Male	
	Crude	Per 100,000 population	Crude	Per 100,000 population	Crude	Per 100,000 population	Crude	Per 100,000 population	Crude	Per 100,000 population	Crude	Per 100,000 population
0-4	350.46 (296.68-490.04)	5.00(4.24-7.00)	440.57 (373.66-620.04)	6.00 (5.09-8.44)	20,973.75	310.20	24,356.98	343.83	21,324.22 (21,270.43-21,463.80)	304.44 (300.20-311.44)	24,797.57(24,730.65-24,977.03)	337.62 (332.53-346.06)
5-9	283.21 (233.81-391.32)	3.97 (3.28-5.49)	375.19 (308.62-517.67)	5.01 (4.12-6.92)	4,201.50	58.46	5,461.96	72.62	4,484.71 (4,435.31-4,592.81)	62.93 (59.65-68.42)	5,837.14 (5,770.58-5,979.62)	78.01 (73.89-84.93)
10-14	288.98 (228.5-387.45)	3.99 (3.15-5.49)	335.80 (266.28-449.61)	4.43 (3.51-5.93)	4,900.87	63.57	6,007.52	74.61	5,189.85 (5,129.37-5288,32)	71.64 (68.49-76.99)	6,343.32 (6,273.79-6,457.13)	83.62 (80.11-89.55)
15-19	552.44 (426.32-727.83)	7.09 (5.47-9.34)	486.38 (376.53-639.32)	6.00 (4.65-7.89)	14,368.91	173.87	14,887.38	173.57	14,921.35 (14,795.22-15,906.74)	191.57 (186.10-200.91)	15,373.76 (15,263.91-15,526.70)	189.70(185.05-197.59)
20-24	1,283.04 (989.71-1,681.11)	15.45 (11.91-20.24)	1,180.43 (903.46-1,547.65)	14.06(10.76-18.43)	25,362.48	301.49	28,403.21	329.21	26,645.52 (26,352.19-15096.74)	320.78 (308.87-341.02)	29,583.64 (29,306.66-29,950.85)	352.37 (341.61-370.80)
25-29	1,664.15 (1,279.36-2,185.46)	20.10 (15.46-26.40)	1,529.13 (1,176.25-2,011.06)	18.72(14.40-24.63)	33,678.38	400.32	45,289.41	531.37	35,342.53 (34,957.73-35,863.83)	426.95 (411.49-453.35)	46,818.54 (46,465.66-47,300.47)	573.32 (558.92-597.95)
30-34	1,817.36 (1,400.31-2,396.65)	21.64 (16.68-28.54)	1,733.41 (1,352.16-2,289.19)	21.21 (16.55-28.01)	54,815.75	643.92	84,709.87	992.99	56,633.11 (56,216.05-57,212.39)	674.47 (657.79-703.01)	86,443.28 (86,062.02-86,999.06)	1,057.85 (1,041.30-1,085.86)
35-39	1,967.91 (1,530.12-2,596.88)	22.81 (17.73-30.09)	1,894.48 (1,491.57-2,516.14)	22.92 (18.05-30.44)	85,610.62	975.43	132,105.11	1,519.06	87,578.54 (87,140.73-88,207.49)	1,014.94 (997.21-1,045.03)	133,999.6 (133,596.68-134,621.25)	1,621.27 (1,603.22-1,651.71)
40-44	1,882.91 (1,467.20-2,491.13)	23.49 (18.31-31.08)	1,827.07 (1,453.83-2,436.42)	24.17 (19.23-32.23)	112,345.02	1,418.88	181,981.18	2,348.91	114,227.93 (113,812.21-114,836.14)	1,425.19 (1,406.88-1,456.27)	183,808.25 (183,435.00-184,417.59)	2,431.68 (2,412.45-2,463.91)
45-49	1,575.09 (1,240.08-2,093.94)	22.43 (17.66-29.82)	1,555.38 (1,261.94-2,098.43)	23.82 (19.33-32.37)	136,010.39	1,948.05	225,365.80	3,350.59	137,585.49 (137,250.47-138,104.32)	1,959.40 (1,941.74-1,989.22)	226,921.18 (226,627.73-227,464.22)	3,475.06 (3,455.73-3,507.20)
50-54	1,412.74 (1,124.79-1,894.26)	21.61 (17.21-28.98)	1,417.50 (1,161.01-1,923.33)	23.85 (19.54-32.37)	170,469.01	2,653.63	280,891.39	4,632.03	171,881.75 (171,593.80-172,363.26)	2,629.16 (2,611.95-2,658.14)	282,308.89 (282,052.39-282,814.72)	4,750.82 (4,731.28-4,783.19)
55-59	1,283.13 (1,041.9-1,735.07)	21.44 (17.41-28.99)	1,320.88 (1,104.39-1,804.19)	24.90 (20.82-34.01)	216,891.24	3,691.15	349,567.79	6,473.43	218,174.37 (217,933.13-218,626.30)	3,645,67 (3,628.26-3,674.66)	350,888.67 (350,672.18-351,371.98)	6,614.25 (6,593.43-6,648.26)
60-64	1,060.56 (882.79-1,447.61)	21.08 (17.54-28.77)	1,142.95 (972.8-1,568.26)	26.20 (22.30-35.95)	265,262.15	5,379.19	405,063.28	9,254.17	266,322.71 (266,144.94-266,709.75)	5,292.72 (5,275.18-5,321.49)	406,206.23 (406,036.07-406,631.53)	9,312.21 (9,289.91-9,348.16)
65-69	876.96 (748.9-1,212.51)	22.00 (18.79-30.42)	991.73 (863.92-1,373.64)	29.68 (25.85-41.11)	276,557.56	7,073.61	424,119.99	12,775.13	277,434.52 (277,306.46-277,770.06)	6.961.10 (6,942.31-6,991.52)	425,111.72 (424,983.91-425,493.63)	12,721.79 (12,695.94-12,762.90)
70-74	717.94 (630.53-1,002.1)	24.16 (21.22-33.72)	812.17 (719.78-1,130.21)	33.84 (29.99-47.09)	254,244.97	8,777.73	373,054.42	16,129.05	254,962.91 (254,875.50-255,247.07)	8,579.76 (8,558.54-8,613.48)	373,866.59 (373,774.20-374,184.62)	15,578.17 (15,548.18-15,625.26)
75-79	570.68 (512.28-803.48)	27.62 (24.79-38.89)	615.35 (555.38-858.12)	39.68 (35.81-55.33)	208,012.95	10,542.01	277,247.97	19,272.58	208,583.63 (208,525.23-208,816.43)	10,095.95 (10,070.26-10,133.94)	277,863.32 (277,803.35-278,106.09)	17,916.49 (17,880.68-17,971.82)
80-84	479.30 (439.52-682.54)	33.91 (31.10-48.29)	467.10 (427.42-659.36)	48.80 (44.65-68.88)	164,451.44	12,313.65	191,233.38	21,817.22	164,930.74 (164,890.95-165,133.97)	11,669.90 (11,638.80-11,718.19)	191,700.48 (191,660.79-191,892.73)	20,027.00 (19,982.35-20,095.88)
85-89	346.82 (322.39-497.67)	44.73 (41.58-64.18)	291.28 (267.69-414.38)	64.23 (59.03-91.37)	100,221.50	13,506.01	98,113.57	22,594.06	100,568.32 (100,543.88-100,719.16)	12,970.18 (12,928.60-13,034.36)	98,404.85 (98,381.26-98,527.94)	21,698.02 (21,638.99-21,789.39)
90 +	269.93 (249.99-392.83)	60.39 (55.93-87.89)	181.14 (166.58-261.31)	81.12 (74.59-117.01)	63,817.72	12,617.76	48,447.41	20,404.75	64,087.66 (64,067.71-64,210.55)	14,338.31 (14,282.38-14,426.20)	48,628.56 (48,614.00-48,708.73)	21,775.28 (21,700.69-21,892.29)
Total	18,683.62 (15,045.19-25,109.86)	422.91 (359.46-583.48)	18,597.96 (15,203.26-25,118.34)	518.64 (448.27-718.18)	2,212,196.23	82,848.93	3,196,307.63	143,089.18	2,230,879.86 (2,227,241.41-2,237,306.08)	82,634.16 (82,274.70-83,217.64)	3,214,905.59 (3,211,510.90-3,221,425.98)	140,594.53 (140,146.26-141,312.71)

*Estimated mean (95% Uncertainty Interval).

A total of 37,281 years of life were lost due to disability (i.e., YLD), accounting for just 0.69% of the total DALY burden. Mild to moderate cases contributed to 26.55% of the crude YLD, long covid to 42.46%, severe cases to 21.76% and critical to 9.22% (Table 3). In contrast, severe cases were responsible for most the largest fraction of the YLD rate (approx. 38% of the YLD rate) (Table 3). Deaths due to COVID-19 resulted in 5,408,504 years of life lost due to premature mortality (i.e., YLL), with males accounting for 59% of the YLL (Table 3). The COVID-19 deaths resulted, on average, in 24 years of life lost due to premature mortality.

**Fig 2:**
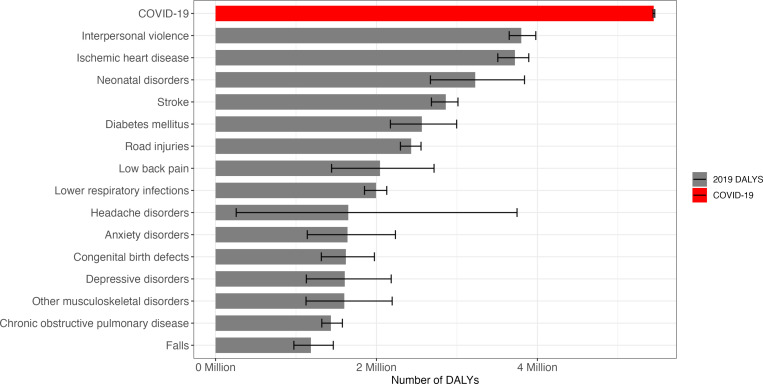
Estimate of the number of DALYs lost for COVID-19 in 2020 and from the pre-pandemic 15 leading causes of disease and injury in Brazil in 2019.

When comparing the estimated DALYs resulting from COVID-19 in 2020 with Brazil’s leading causes of disease and injury in 2019, COVID-19’s burden surpassed the burden of all diseases and injuries, suggesting that it may have been the leading cause of disease and injury in Brazil in 2020 (Fig 2). The estimated COVID-19 DALYs were considerably greater than those from interpersonal violence (3,649,901 to 3,979,773 DALYs) and ischemic heart disease (3,507,748 to 3,892,657 DALYs).

**Fig 3:**
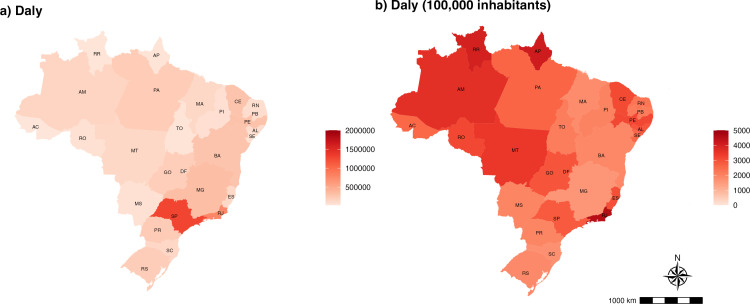
Distribution of estimated (A) Disability-adjusted life years (DALYs) and (B) DALYs per 100,000 populations due to COVID-19 in Brazil by the state of residence between February 26, 2020, and December 31, 2020.

Fig 3 illustrates the distribution of DALYs due to COVID-19 in the Brazilian states in 2020. More DALYs were lost in the Southeast states of São Paulo (1,285,542 DALYs), Rio de Janeiro (775,737 DALYs), and Minas Gerais (332,090 DALYs), while the states of Acre (22,030 DALYs), Roraima (25,519 DALYs), and Amapá (32,251 DALYs) presented the lowest estimates (Fig 3a and S3 Table). However, when accounting for the state’s population size, we note that the highest COVID-19 burden was experienced Rio de Janeiro (4,504 DALYs), followed by states of the North of the country, such as Amapá (4,106 DALYs), Roraima (3,981 DALYs) and Amazonas (3,638 DALYs) (Fig 3b and S3 Table).

## Discussion

We estimated that the COVID-19 burden in Brazil reached around 5 million DALYs lost in 2020. Both DALYs and DALYs per 100,000 persons were higher in males than females. These findings align with estimates from the US [39], Germany [40], Italy [41] and Ireland [28]. Although COVID-19 cases data show similar numbers of cases between males and females, there appear to be gender differences in vulnerability to severe disease and death [42–46]. Assuming there were no major changes in the disease burden experienced by the Brazilian population from 2019 to 2020, our results showed that COVID-19 led to enough DALYs lost to rank as the leading cause of disease and injury in the country in 2020. The COVID-19 burden was higher than all 15 leading causes of disease and injury as estimated by the GBD 2019, including interpersonal violence, ischemic heart disease, neonatal disorders, and stroke. Most of the COVID-19 health impact was due to premature mortality, representing 99.31% of the DALYs.

These effects remain consistent across all our sensitivity analyses, which intended to accommodate a range of scenarios regarding over- and under-reporting of mild to moderate, severe, and critical COVID-19 cases as well as long covid cases. Even in the most conservative scenario, the burden produced by COVID-19 in 2020 remained the leading cause of DALYs lost in Brazil. The results from the sensitivity and scenario analyses also highlight that variations in the inputs used to calculate the YLD have a minor impact on the overall DALYs estimates as YLD only contribute to a small percentage of the total DALYs.

To date, several studies have estimated the COVID-19 burden in 2020 in different countries [31,32,39–41,43–48]. Unfortunately, the use of different methodologies poses barriers to the direct comparison among the estimates. With that in mind, we can still compare our results to those from six studies (India[46], Netherlands[32], Germany[40], Malta[49], Denmark[31], Scotland[27]). Results presented in these studies corroborate our findings regarding the overwhelming contribution of the YLL in the DALYs estimates with the burden derived from YLL, ranging from 95% in Malta to 99,6% in India[46,49]. Furthermore, the high relative contribution of YLL in the COVID-19 disease burden is in line with what was observed in the GBD study estimates of the relative contribution of YLL from lower respiratory infections [23,24]. YLL tends to outweight YLD due a combination of factors, including as methodological influences, population context, disease impact and healthcare access and quality. YLL is calculated based on the number of deaths and the age at which they occur, with greater weight given to deaths occurring at young ages. For diseases with high mortality, such as COVID-19 in vulnerable populations (i.e., non-vaccinated), YLL often predominates in the DALY calculation [50].

As expected, the crude estimates reflected the underlying countries’ population, with India reporting the higher crude estimates (approximately 14 million DALYs) and Malta the lowest (5478 DALYs). In contrast, regarding the DALY rate, findings from Scotland had shown the highest per capita burden (ranging from 1770 to 1980 DALY/100,000), and those from Germany the lowest (368 DALY/100,000) [27,41]. Adding our results to this body of knowledge suggests that the COVID-19 burden in Brazil (i.e., 2,567 DALY/100,000) was higher than in any other country. Such differences between countries reflect not only how hard the epidemic hit each country but also its population structure, the age distribution of the outcomes (especially the deaths), data availability, data assumptions, and model choices. Although comparisons in the COVID-19 DALYs can be useful to demonstrate the extent to which each country was affected, care must be taken when interpreting such results, given the difference in the timing of the peak of cases, especially regarding the absolute rate difference which might not be the most informative or appropriate, given that baseline vulnerability in each country will significantly differ.

Previous studies have highlighted the local and regional variations in COVID-19’s impact on population health. Initial estimates of COVID-19 excess deaths indicated that the pandemic initially hit larger cities in Southeast Brazil hardest, with patterns evolving as the pandemic progressed [51]. Another study on the burden averted by vaccination revealed that vaccination saved more lives in the North of Brazil, where the incidence rates were higher [10]. Numerous studies have reported disproportionally high hospitalisation and mortality rates in Brazil’s North region. Consequently, the greatest decline in life expectancy at birth in Brazil was estimated for states in this region. The elevated COVID-19 burden estimated in the present analysis likely results from the two COVID-19 waves that affected the region [7,9–11,52,53]. Regarding the regional differences, the decentralisation of actions is a fundamental component of the Brazilian Health System (*Sistema Único de Saúde*, SUS). This allowed states to independently adapt their protective measures, such as social distancing, isolation, lockdowns, and other restrictions, as well as testing protocols. Consequently, this variability makes it challenging to account for the differences implemented by each state.[54].

This is the first study to provide comprehensive estimates of the COVID-19 burden of disease in Brazil. To this end, we adopted a widely used protocol developed for appropriate estimation of DALYs due to a disease or injury [23–29,31,32,39–41,45–47]. Nevertheless, our study has some limitations. Our estimates directly depend on the quality of surveillance registries that correspond to data from more than three years ago. However, the databases we used correspond to the best available evidence on COVID-19 cases and deaths and were largely used in various studies in Brazil [6–11,51–53,55–61]. Further, we lack sufficient data to estimate the COVID-19 burden for the following years, especially due to the absence of covariates such as vaccination and variants over the entire course of the pandemic in Brazil. Unfortunately, the databases used do not present information regarding the variant causing the COVID-19 episode or the individual vaccination status when presenting the disease. When comparing the DALY estimates with pre-pandemic causes, there are some potential drawbacks to take into account, especially related to the competing risk of death. It is unlikely that all COVID-19 deaths are additional, and it is likely that at least part of those deaths replace deaths that would have occurred due to other causes. The extent to which this is true should not be overstated [62]. Studies have shown that in 2020, there was a reduction of 8.8% in ischemic heart disease mortality compared to the previous year [63]. Nevertheless, given that the DALY difference between ischemic heart disease and COVID-19 was 32%, it is unlikely that this reduction would change our conclusions on the enormous COVID-19 burden. Our estimates are based on a published consensus method developed by the European Burden of Disease Network and the European Centre for Disease Control and Prevention (ECDC) [64]. However, the estimates of the number of cases and duration of long covid remain highly uncertain [30,65–67]. Our sensitivity analyses showed that a wide range of assumptions had a minimal impact on the overall burden of COVID-19, given that YLD contributed to less than 1% of the total DALYs lost. However, as more epidemiological information on long-COVID emerges, it should be integrated into the modelling process to increase the robustness of the YLD estimates.

We have shown that the direct impact of the COVID-19 pandemic on the Brazilian population’s health has been substantial. Despite the mitigation efforts, in 2020, the disease stood out as the leading cause of DALYs relative to all other health conditions observed in the previous year. Future work should be directed towards international comparison over longer periods, incorporating the role of vaccination rollout and the upsurge of variants of concern.

## Supporting information

S1 Table Summary of Disability-Adjusted Life Years of the 2019 GBD results for Brazil according to the cause. (PDF)

S2 Table Morbidity sensitivity analysis: impact on COVID-19 YLD, Brazil, 2020. (PDF)

S3 Table Total number of COVID-19 mild, severe and critical cases, long covid, Dalys and Dalys/100,000 person in Brazil by state between February 26, 2020, to December 31, 2020. (PDF)

S1 Text Code used in the analysis of the manuscript “Disability-adjusted life Years associated with COVID-19 in Brazil, 2020”. (PDF)

## Abbreviations

COVID‐19: Coronavirus Disease 2019
DALY: Disability-adjusted life year
ECDC: European Centre for Disease Prevention and Control
YLD: Years of life lost due to disability
YLL: Years of life lost due to premature mortality
ICU: Intensive care unit
GBD: Global Burden of Disease
MoH: Ministry of Health
WHO: World Health Organization
