# Supplementary material for: Disability-adjusted life years associated with COVID-19 in Brazil, 2020
Source: PLoS One. 2025 Mar 27;20(3):e0319941. doi: 10.1371/journal.pone.0319941 (PMC11949356; doi:10.1371/journal.pone.0319941)
Supplement: S1 Table — (PDF) [file pone.0319941.s001.pdf]

S1 Table. Summary of Disability-Adjusted Life Years of the 2019 GBD results for Brazil according to the cause. Available at https://vizhub.healthdata.org/gbd-results/

measure_id	measure_name	location_id	location_name	sex_id	sex_name	age_id	age_name	cause_id	cause_name	metric_id	metric_name	year	val	upper	lower
**2**	DALYs (Disability-Adjusted Life Years)	135	Brazil	3	Both	22	All ages	387	Protein-energy malnutrition	1	Number	2019	234582.05856062743	261251.48035658323	207999.14662085605
**2**	DALYs (Disability-Adjusted Life Years)	135	Brazil	3	Both	22	All ages	388	Iodine deficiency	1	Number	2019	2583.8105458575424	4972.80913686506	1148.815283364424
**2**	DALYs (Disability-Adjusted Life Years)	135	Brazil	3	Both	22	All ages	389	Vitamin A deficiency	1	Number	2019	18072.25162585565	26646.48261649007	11056.565425928065
**2**	DALYs (Disability-Adjusted Life Years)	135	Brazil	3	Both	22	All ages	390	Dietary iron deficiency	1	Number	2019	659743.0313082277	1011720.443781015	424385.7067777086
**2**	DALYs (Disability-Adjusted Life Years)	135	Brazil	3	Both	22	All ages	391	Other nutritional deficiencies	1	Number	2019	27677.945136299604	36330.505881169374	21571.04135807696
**2**	DALYs (Disability-Adjusted Life Years)	135	Brazil	3	Both	22	All ages	393	Sexually transmitted infections excluding HIV	1	Number	2019	69729.49466205647	110940.35582042411	47597.87540351133
**2**	DALYs (Disability-Adjusted Life Years)	135	Brazil	3	Both	22	All ages	400	Acute hepatitis	1	Number	2019	21594.159012802465	27522.933292457652	17149.636314300074
**2**	DALYs (Disability-Adjusted Life Years)	135	Brazil	3	Both	22	All ages	405	Leprosy	1	Number	2019	2514.6243061755026	3715.0250083651167	1598.4564031376567
**2**	DALYs (Disability-Adjusted Life Years)	135	Brazil	3	Both	22	All ages	408	Other unspecified infectious diseases	1	Number	2019	108570.48583819282	149236.4283096354	86236.55235645702
**2**	DALYs (Disability-Adjusted Life Years)	135	Brazil	3	Both	22	All ages	515	Asthma	1	Number	2019	448965.3970649907	661635.6781391872	310381.4920426175
**2**	DALYs (Disability-Adjusted Life Years)	135	Brazil	3	Both	22	All ages	516	Interstitial lung disease and pulmonary sarcoidosis	1	Number	2019	73306.04561488303	88621.33508084709	47761.2723410374
**2**	DALYs (Disability-Adjusted Life Years)	135	Brazil	3	Both	22	All ages	520	Other chronic respiratory diseases	1	Number	2019	66565.39014134654	77148.04844973101	56922.70774256178
**2**	DALYs (Disability-Adjusted Life Years)	135	Brazil	3	Both	22	All ages	521	Cirrhosis and other chronic liver diseases	1	Number	2019	1170623.4306604452	1238023.584805878	1113984.9992971169
**2**	DALYs (Disability-Adjusted Life Years)	135	Brazil	3	Both	22	All ages	718	Self-harm	1	Number	2019	626719.5755263034	682680.3157763474	594617.8013324933
**2**	DALYs (Disability-Adjusted Life Years)	135	Brazil	3	Both	22	All ages	724	Interpersonal violence	1	Number	2019	3799992.1088724015	3979773.1764879986	3649901.7111265394
**2**	DALYs (Disability-Adjusted Life Years)	135	Brazil	3	Both	22	All ages	729	Exposure to forces of nature	1	Number	2019	3167.9132166958893	4075.524075197417	2517.0942352995194

**2**	DALYs (Disability-Adjusted Life Years)	135	Brazil	3	Both	22	All ages	842	Environmental heat and cold exposure	1	Number	2019	105988.62251542149	152789.57393983952	69533.75421468247
**2**	DALYs (Disability-Adjusted Life Years)	135	Brazil	3	Both	22	All ages	843	Ebola	1	Number	2019	0.0	0.0	0.0
**2**	DALYs (Disability-Adjusted Life Years)	135	Brazil	3	Both	22	All ages	630	Low back pain	1	Number	2019	2044102.3335921352	2715670.0489839525	1441813.5374309267
**2**	DALYs (Disability-Adjusted Life Years)	135	Brazil	3	Both	22	All ages	631	Neck pain	1	Number	2019	542596.5167578775	792398.1854230537	356801.6318433749
**2**	DALYs (Disability-Adjusted Life Years)	135	Brazil	3	Both	22	All ages	632	Gout	1	Number	2019	19550.6607715423	28144.978359007106	12433.513086303428
**2**	DALYs (Disability-Adjusted Life Years)	135	Brazil	3	Both	22	All ages	639	Other musculoskeletal disorders	1	Number	2019	1600235.5253439571	2195213.8481951742	1124478.7223276002
**2**	DALYs (Disability-Adjusted Life Years)	135	Brazil	3	Both	22	All ages	641	Congenital birth defects	1	Number	2019	1619653.8168811353	1974399.0475665892	1314928.7156885008
**2**	DALYs (Disability-Adjusted Life Years)	135	Brazil	3	Both	22	All ages	716	Other unintentional injuries	1	Number	2019	242136.55680485748	279349.45549032866	211359.1858333827
**2**	DALYs (Disability-Adjusted Life Years)	135	Brazil	3	Both	22	All ages	338	Diphtheria	1	Number	2019	110.85583053911412	152.64442798078986	82.10256686693408
**2**	DALYs (Disability-Adjusted Life Years)	135	Brazil	3	Both	22	All ages	339	Whooping cough	1	Number	2019	8179.941856031956	11161.983075938499	5890.61650961598
**2**	DALYs (Disability-Adjusted Life Years)	135	Brazil	3	Both	22	All ages	340	Tetanus	1	Number	2019	4130.272460780992	10851.533084507813	2764.7388447657404
**2**	DALYs (Disability-Adjusted Life Years)	135	Brazil	3	Both	22	All ages	341	Measles	1	Number	2019	66.18629233612604	108.52209412358579	49.370643500290065
**2**	DALYs (Disability-Adjusted Life Years)	135	Brazil	3	Both	22	All ages	411	Esophageal cancer	1	Number	2019	323233.2571911268	340083.85491740744	306838.13952648477
**2**	DALYs (Disability-Adjusted Life Years)	135	Brazil	3	Both	22	All ages	414	Stomach cancer	1	Number	2019	545219.2359096949	568478.4945809729	519168.1577913597
**2**	DALYs (Disability-Adjusted Life Years)	135	Brazil	3	Both	22	All ages	417	Liver cancer	1	Number	2019	139930.43347273007	147341.30774208912	132617.60506465824
**2**	DALYs (Disability-Adjusted Life Years)	135	Brazil	3	Both	22	All ages	423	Larynx cancer	1	Number	2019	146503.07029315343	153963.2905853141	138630.09397477887
**2**	DALYs (Disability-Adjusted Life Years)	135	Brazil	3	Both	22	All ages	426	Tracheal, bronchus, and lung cancer	1	Number	2019	866748.9304611183	906754.520746419	824500.8678500055

**2**	DALYs (Disability-Adjusted Life Years)	135	Brazil	3	Both	22	All ages	1022	Other malignant neoplasms	1	Number	2019	388650.352683842	419576.8905049676	365380.3954373924
**2**	DALYs (Disability-Adjusted Life Years)	135	Brazil	3	Both	22	All ages	1023	Other cardiovascular and circulatory diseases	1	Number	2019	293877.3304039161	323538.2812394983	268964.4652352467
**2**	DALYs (Disability-Adjusted Life Years)	135	Brazil	3	Both	22	All ages	529	Appendicitis	1	Number	2019	47947.471110332	56548.60264096979	36292.34338771591
**2**	DALYs (Disability-Adjusted Life Years)	135	Brazil	3	Both	22	All ages	530	Paralytic ileus and intestinal obstruction	1	Number	2019	175523.93493341957	195209.8728150531	146394.13854217678
**2**	DALYs (Disability-Adjusted Life Years)	135	Brazil	3	Both	22	All ages	531	Inguinal, femoral, and abdominal hernia	1	Number	2019	153769.20006026153	196532.97701761752	119238.87647589402
**2**	DALYs (Disability-Adjusted Life Years)	135	Brazil	3	Both	22	All ages	532	Inflammatory bowel disease	1	Number	2019	46349.59919362761	54190.49827224871	39647.058057770024
**2**	DALYs (Disability-Adjusted Life Years)	135	Brazil	3	Both	22	All ages	533	Vascular intestinal disorders	1	Number	2019	89266.65842071164	96308.73550847203	82617.83716337186
**2**	DALYs (Disability-Adjusted Life Years)	135	Brazil	3	Both	22	All ages	534	Gallbladder and biliary diseases	1	Number	2019	339869.1651266341	436556.5890696655	265834.5593248367
**2**	DALYs (Disability-Adjusted Life Years)	135	Brazil	3	Both	22	All ages	535	Pancreatitis	1	Number	2019	172622.08638568	185958.96910009126	153155.2111428051
**2**	DALYs (Disability-Adjusted Life Years)	135	Brazil	3	Both	22	All ages	619	Endocrine, metabolic, blood, and immune disorders	1	Number	2019	531395.3049454737	638577.2962763697	401818.75219737354
**2**	DALYs (Disability-Adjusted Life Years)	135	Brazil	3	Both	22	All ages	627	Rheumatoid arthritis	1	Number	2019	103504.867308888	133453.7900765186	75336.61944832184
**2**	DALYs (Disability-Adjusted Life Years)	135	Brazil	3	Both	22	All ages	628	Osteoarthritis	1	Number	2019	497542.53737190366	985231.3739572625	249691.8197123114
**2**	DALYs (Disability-Adjusted Life Years)	135	Brazil	3	Both	22	All ages	297	Tuberculosis	1	Number	2019	213105.26705559008	227018.34393998983	200796.92787784542
**2**	DALYs (Disability-Adjusted Life Years)	135	Brazil	3	Both	22	All ages	298	HIV/AIDS	1	Number	2019	807432.7042503263	849402.5665427356	773530.5753850581
**2**	DALYs (Disability-Adjusted Life Years)	135	Brazil	3	Both	22	All ages	302	Diarrheal diseases	1	Number	2019	565446.014935547	683841.2887416611	468026.2268654036
**2**	DALYs (Disability-Adjusted Life Years)	135	Brazil	3	Both	22	All ages	321	Other intestinal infectious diseases	1	Number	2019	1226.1867528835137	1943.0997750385575	675.4465484899882
**2**	DALYs (Disability-Adjusted Life Years)	135	Brazil	3	Both	22	All ages	322	Lower respiratory infections	1	Number	2019	1994790.0108112518	2127570.6090563494	1851232.561746713

**2**	DALYs (Disability-Adjusted Life Years)	135	Brazil	3	Both	22	All ages	328	Upper respiratory infections	1	Number	2019	230934.93604442215	359653.16783655185	141602.31330491856
**2**	DALYs (Disability-Adjusted Life Years)	135	Brazil	3	Both	22	All ages	329	Otitis media	1	Number	2019	59849.819711779186	97455.24470788451	35440.79787689351
**2**	DALYs (Disability-Adjusted Life Years)	135	Brazil	3	Both	22	All ages	332	Meningitis	1	Number	2019	129800.01661433573	145349.80547612556	114468.24148970917
**2**	DALYs (Disability-Adjusted Life Years)	135	Brazil	3	Both	22	All ages	337	Encephalitis	1	Number	2019	27246.293182188376	31609.680076488672	20267.89821030364
**2**	DALYs (Disability-Adjusted Life Years)	135	Brazil	3	Both	22	All ages	364	Food-borne trematodiases	1	Number	2019	0.0	0.0	0.0
**2**	DALYs (Disability-Adjusted Life Years)	135	Brazil	3	Both	22	All ages	365	Other neglected tropical diseases	1	Number	2019	51342.86937014688	72466.74108800195	34243.73399672275
**2**	DALYs (Disability-Adjusted Life Years)	135	Brazil	3	Both	22	All ages	366	Maternal disorders	1	Number	2019	130803.15596251882	140839.12150293926	121016.18324467364
**2**	DALYs (Disability-Adjusted Life Years)	135	Brazil	3	Both	22	All ages	429	Breast cancer	1	Number	2019	598058.7447726616	636738.9304932614	563114.2783994399
**2**	DALYs (Disability-Adjusted Life Years)	135	Brazil	3	Both	22	All ages	432	Cervical cancer	1	Number	2019	348416.2536160296	404298.3808616996	324215.10040369886
**2**	DALYs (Disability-Adjusted Life Years)	135	Brazil	3	Both	22	All ages	435	Uterine cancer	1	Number	2019	74322.142068717	79212.04087847899	69589.60697801026
**2**	DALYs (Disability-Adjusted Life Years)	135	Brazil	3	Both	22	All ages	438	Prostate cancer	1	Number	2019	413225.1886873036	605638.5953097729	359721.3260855095
**2**	DALYs (Disability-Adjusted Life Years)	135	Brazil	3	Both	22	All ages	441	Colon and rectum cancer	1	Number	2019	644732.2504714219	672419.4198270678	611425.9615737835
**2**	DALYs (Disability-Adjusted Life Years)	135	Brazil	3	Both	22	All ages	444	Lip and oral cavity cancer	1	Number	2019	148871.92349832004	157204.7166435582	141013.9021338604
**2**	DALYs (Disability-Adjusted Life Years)	135	Brazil	3	Both	22	All ages	447	Nasopharynx cancer	1	Number	2019	18249.4267227175	19339.822884608195	17075.063229832744
**2**	DALYs (Disability-Adjusted Life Years)	135	Brazil	3	Both	22	All ages	450	Other pharynx cancer	1	Number	2019	109752.96794941707	117179.92693370876	102718.4402106868
**2**	DALYs (Disability-Adjusted Life Years)	135	Brazil	3	Both	22	All ages	541	Other digestive diseases	1	Number	2019	131268.78778671002	169138.56680228756	95652.62353742278
**2**	DALYs (Disability-Adjusted Life Years)	135	Brazil	3	Both	22	All ages	543	Alzheimer's disease and other dementias	1	Number	2019	855327.6514646594	1874372.874799152	377333.1253174232

**2**	DALYs (Disability-Adjusted Life Years)	135	Brazil	3	Both	22	All ages	544	Parkinson's disease	1	Number	2019	159998.24812870624	173337.43011335985	145339.4958632942
**2**	DALYs (Disability-Adjusted Life Years)	135	Brazil	3	Both	22	All ages	545	Idiopathic epilepsy	1	Number	2019	404151.5433089046	549563.1036406613	287850.0066736286
**2**	DALYs (Disability-Adjusted Life Years)	135	Brazil	3	Both	22	All ages	546	Multiple sclerosis	1	Number	2019	24794.756487326107	31459.440766283948	19863.57294936732
**2**	DALYs (Disability-Adjusted Life Years)	135	Brazil	3	Both	22	All ages	342	Varicella and herpes zoster	1	Number	2019	21686.22184558426	29505.478869269034	15177.04198318113
**2**	DALYs (Disability-Adjusted Life Years)	135	Brazil	3	Both	22	All ages	345	Malaria	1	Number	2019	11097.042138762356	22964.825473702058	5413.096895500839
**2**	DALYs (Disability-Adjusted Life Years)	135	Brazil	3	Both	22	All ages	346	Chagas disease	1	Number	2019	174194.2242029733	302974.3817183899	109039.60418755852
**2**	DALYs (Disability-Adjusted Life Years)	135	Brazil	3	Both	22	All ages	347	Leishmaniasis	1	Number	2019	67617.83450630598	266026.3240235446	2870.27288551281
**2**	DALYs (Disability-Adjusted Life Years)	135	Brazil	3	Both	22	All ages	350	African trypanosomiasis	1	Number	2019	0.0	0.0	0.0
**2**	DALYs (Disability-Adjusted Life Years)	135	Brazil	3	Both	22	All ages	380	Neonatal disorders	1	Number	2019	3227621.9515293366	3840013.8518732428	2670497.9301191354
**2**	DALYs (Disability-Adjusted Life Years)	135	Brazil	3	Both	22	All ages	493	Ischemic heart disease	1	Number	2019	3721023.4544533067	3892657.2285360475	3507748.043631261
**2**	DALYs (Disability-Adjusted Life Years)	135	Brazil	3	Both	22	All ages	494	Stroke	1	Number	2019	2861723.2406806713	3012805.91798038	2683069.8825876014
**2**	DALYs (Disability-Adjusted Life Years)	135	Brazil	3	Both	22	All ages	498	Hypertensive heart disease	1	Number	2019	558147.3630457986	769792.9470385057	502264.9515933891
**2**	DALYs (Disability-Adjusted Life Years)	135	Brazil	3	Both	22	All ages	499	Cardiomyopathy and myocarditis	1	Number	2019	545772.4025089525	621356.7525456359	484988.71270660765
**2**	DALYs (Disability-Adjusted Life Years)	135	Brazil	3	Both	22	All ages	500	Atrial fibrillation and flutter	1	Number	2019	230116.28937971775	279885.89947355114	189167.01024767716
**2**	DALYs (Disability-Adjusted Life Years)	135	Brazil	3	Both	22	All ages	554	Motor neuron disease	1	Number	2019	43968.35832636242	46922.62958930152	40197.573334657085
**2**	DALYs (Disability-Adjusted Life Years)	135	Brazil	3	Both	22	All ages	557	Other neurological disorders	1	Number	2019	140842.80214724535	172541.1166931995	114756.52927059708
**2**	DALYs (Disability-Adjusted Life Years)	135	Brazil	3	Both	22	All ages	559	Schizophrenia	1	Number	2019	431051.70887983096	547015.259264056	313386.82771435555

**2**	DALYs (Disability-Adjusted Life Years)	135	Brazil	3	Both	22	All ages	560	Alcohol use disorders	1	Number	2019	1051583.0262254274	1337046.8849659949	831634.6604850595
**2**	DALYs (Disability-Adjusted Life Years)	135	Brazil	3	Both	22	All ages	561	Drug use disorders	1	Number	2019	467981.9459190504	619187.0331036766	336020.82108711463
**2**	DALYs (Disability-Adjusted Life Years)	135	Brazil	3	Both	22	All ages	654	Dermatitis	1	Number	2019	282390.20230380597	440372.9706626824	169181.34178468125
**2**	DALYs (Disability-Adjusted Life Years)	135	Brazil	3	Both	22	All ages	501	Aortic aneurysm	1	Number	2019	231484.29319789313	245471.0611702248	216550.5087578073
**2**	DALYs (Disability-Adjusted Life Years)	135	Brazil	3	Both	22	All ages	502	Peripheral artery disease	1	Number	2019	54103.231028389935	92661.80924332066	29178.66896367352
**2**	DALYs (Disability-Adjusted Life Years)	135	Brazil	3	Both	22	All ages	503	Endocarditis	1	Number	2019	77155.36132857612	92952.81323205176	58867.27425067627
**2**	DALYs (Disability-Adjusted Life Years)	135	Brazil	3	Both	22	All ages	504	Non-rheumatic valvular heart disease	1	Number	2019	103773.7694791369	110719.99769803298	96149.31340739889
**2**	DALYs (Disability-Adjusted Life Years)	135	Brazil	3	Both	22	All ages	509	Chronic obstructive pulmonary disease	1	Number	2019	1434008.7060512628	1576595.4206850396	1320384.7570499734
**2**	DALYs (Disability-Adjusted Life Years)	135	Brazil	3	Both	22	All ages	510	Pneumoconiosis	1	Number	2019	19338.35357611467	22689.918219180596	16723.97621983232
**2**	DALYs (Disability-Adjusted Life Years)	135	Brazil	3	Both	22	All ages	594	Urinary diseases and male infertility	1	Number	2019	467097.99367524916	517309.5272215422	343967.8542967998
**2**	DALYs (Disability-Adjusted Life Years)	135	Brazil	3	Both	22	All ages	351	Schistosomiasis	1	Number	2019	68482.08483341704	111077.89955477891	42318.819919410256
**2**	DALYs (Disability-Adjusted Life Years)	135	Brazil	3	Both	22	All ages	352	Cysticercosis	1	Number	2019	82940.27211321458	124414.31122835567	51677.211658392895
**2**	DALYs (Disability-Adjusted Life Years)	135	Brazil	3	Both	22	All ages	353	Cystic echinococcosis	1	Number	2019	317.861032060771	453.19621349566603	193.75421317949426
**2**	DALYs (Disability-Adjusted Life Years)	135	Brazil	3	Both	22	All ages	354	Lymphatic filariasis	1	Number	2019	6899.536840284492	10341.38730096891	4416.520015832443
**2**	DALYs (Disability-Adjusted Life Years)	135	Brazil	3	Both	22	All ages	355	Onchocerciasis	1	Number	2019	0.0	0.0	0.0
**2**	DALYs (Disability-Adjusted Life Years)	135	Brazil	3	Both	22	All ages	356	Trachoma	1	Number	2019	121.76387708353211	199.62821500399755	69.60587520686919
**2**	DALYs (Disability-Adjusted Life Years)	135	Brazil	3	Both	22	All ages	357	Dengue	1	Number	2019	40600.26081353127	50448.819078768465	27017.685550875325

**2**	DALYs (Disability-Adjusted Life Years)	135	Brazil	3	Both	22	All ages	358	Yellow fever	1	Number	2019	266.5561584750066	619.1216358101663	94.81405774418437
**2**	DALYs (Disability-Adjusted Life Years)	135	Brazil	3	Both	22	All ages	359	Rabies	1	Number	2019	76.1144898816029	91.6788750583807	64.32282716271003
**2**	DALYs (Disability-Adjusted Life Years)	135	Brazil	3	Both	22	All ages	360	Intestinal nematode infections	1	Number	2019	23911.071584782305	38907.07992797718	13799.149979774564
**2**	DALYs (Disability-Adjusted Life Years)	135	Brazil	3	Both	22	All ages	453	Gallbladder and biliary tract cancer	1	Number	2019	119382.47268703078	137705.40873721457	103997.56464964313
**2**	DALYs (Disability-Adjusted Life Years)	135	Brazil	3	Both	22	All ages	456	Pancreatic cancer	1	Number	2019	339640.9287075585	358273.90454937646	318056.22606638714
**2**	DALYs (Disability-Adjusted Life Years)	135	Brazil	3	Both	22	All ages	459	Malignant skin melanoma	1	Number	2019	69529.99257046377	102738.31474373385	57882.86983172882
**2**	DALYs (Disability-Adjusted Life Years)	135	Brazil	3	Both	22	All ages	462	Non-melanoma skin cancer	1	Number	2019	52194.92329567882	55221.366502837554	47105.490040316836
**2**	DALYs (Disability-Adjusted Life Years)	135	Brazil	3	Both	22	All ages	655	Psoriasis	1	Number	2019	152913.86255285243	201484.67892111622	108428.90491496662
**2**	DALYs (Disability-Adjusted Life Years)	135	Brazil	3	Both	22	All ages	658	Scabies	1	Number	2019	243672.89161035736	385832.5630450454	135745.01328953504
**2**	DALYs (Disability-Adjusted Life Years)	135	Brazil	3	Both	22	All ages	659	Fungal skin diseases	1	Number	2019	100254.32927249909	209874.26794227568	40455.761808981646
**2**	DALYs (Disability-Adjusted Life Years)	135	Brazil	3	Both	22	All ages	660	Viral skin diseases	1	Number	2019	89859.20418004938	134381.24744126888	57550.48405588331
**2**	DALYs (Disability-Adjusted Life Years)	135	Brazil	3	Both	22	All ages	661	Acne vulgaris	1	Number	2019	85565.91442653604	138064.8124070577	51880.41247180435
**2**	DALYs (Disability-Adjusted Life Years)	135	Brazil	3	Both	22	All ages	662	Alopecia areata	1	Number	2019	16568.072284319805	24457.41286006575	10492.02308560561
**2**	DALYs (Disability-Adjusted Life Years)	135	Brazil	3	Both	22	All ages	663	Pruritus	1	Number	2019	20627.271669586804	36857.97003755061	9870.944673201202
**2**	DALYs (Disability-Adjusted Life Years)	135	Brazil	3	Both	22	All ages	664	Urticaria	1	Number	2019	103529.62717924714	149034.29360992106	67672.03790801256
**2**	DALYs (Disability-Adjusted Life Years)	135	Brazil	3	Both	22	All ages	665	Decubitus ulcer	1	Number	2019	33550.7079784717	43404.76218791138	16556.15339908274
**2**	DALYs (Disability-Adjusted Life Years)	135	Brazil	3	Both	22	All ages	668	Other skin and subcutaneous diseases	1	Number	2019	117657.54753880178	209098.4233846312	59926.97313489101

**2**	DALYs (Disability-Adjusted Life Years)	135	Brazil	3	Both	22	All ages	854	Executions and police conflict	1	Number	2019	70181.58973609358	90602.48351118006	49421.09536509048
**2**	DALYs (Disability-Adjusted Life Years)	135	Brazil	3	Both	22	All ages	935	Zika virus	1	Number	2019	92.37676982892964	139.20064298851582	56.161400532012216
**2**	DALYs (Disability-Adjusted Life Years)	135	Brazil	3	Both	22	All ages	936	Guinea worm disease	1	Number	2019	0.0	0.0	0.0
**2**	DALYs (Disability-Adjusted Life Years)	135	Brazil	3	Both	22	All ages	945	Conflict and terrorism	1	Number	2019	251.94153894619757	412.4405087321522	157.7948716120282
**2**	DALYs (Disability-Adjusted Life Years)	135	Brazil	3	Both	22	All ages	603	Gynecological diseases	1	Number	2019	740661.3216003797	1036025.3097278355	509349.9377924529
**2**	DALYs (Disability-Adjusted Life Years)	135	Brazil	3	Both	22	All ages	613	Hemoglobinopathies and hemolytic anemias	1	Number	2019	174195.14954804795	222334.6475540035	140207.8222781778
**2**	DALYs (Disability-Adjusted Life Years)	135	Brazil	3	Both	22	All ages	695	Other transport injuries	1	Number	2019	90285.57926538092	95607.87070020013	84592.47780294144
**2**	DALYs (Disability-Adjusted Life Years)	135	Brazil	3	Both	22	All ages	697	Falls	1	Number	2019	1185461.1364943713	1463438.2039067405	973367.0010282937
**2**	DALYs (Disability-Adjusted Life Years)	135	Brazil	3	Both	22	All ages	698	Drowning	1	Number	2019	347221.11798888486	370316.1721748674	322317.4618871036
**2**	DALYs (Disability-Adjusted Life Years)	135	Brazil	3	Both	22	All ages	699	Fire, heat, and hot substances	1	Number	2019	140710.84078678858	191618.91874224992	106379.31892404404
**2**	DALYs (Disability-Adjusted Life Years)	135	Brazil	3	Both	22	All ages	700	Poisonings	1	Number	2019	14867.173487134256	17172.437089642593	12908.755743976704
**2**	DALYs (Disability-Adjusted Life Years)	135	Brazil	3	Both	22	All ages	704	Exposure to mechanical forces	1	Number	2019	263249.8794019479	325173.29596113676	217115.12293279546
**2**	DALYs (Disability-Adjusted Life Years)	135	Brazil	3	Both	22	All ages	708	Adverse effects of medical treatment	1	Number	2019	87980.44904404019	95855.74051960585	73209.15284976117
**2**	DALYs (Disability-Adjusted Life Years)	135	Brazil	3	Both	22	All ages	709	Animal contact	1	Number	2019	34186.948167399445	41480.720018169	29121.95098990557
**2**	DALYs (Disability-Adjusted Life Years)	135	Brazil	3	Both	22	All ages	712	Foreign body	1	Number	2019	230239.96919437422	263996.2268414257	199331.8529069933
**2**	DALYs (Disability-Adjusted Life Years)	135	Brazil	3	Both	22	All ages	980	Bacterial skin diseases	1	Number	2019	139272.23884671362	178352.73475194775	70739.77970530563
**2**	DALYs (Disability-Adjusted Life Years)	135	Brazil	3	Both	22	All ages	981	Blindness and vision loss	1	Number	2019	700376.728553627	950927.9798751222	490549.93816533516

**2**	DALYs (Disability-Adjusted Life Years)	135	Brazil	3	Both	22	All ages	992	Upper digestive system diseases	1	Number	2019	488715.04932539567	739876.352950235	329458.09373301954
**2**	DALYs (Disability-Adjusted Life Years)	135	Brazil	3	Both	22	All ages	465	Ovarian cancer	1	Number	2019	150453.52234241	163533.06304772856	138189.26598279134
**2**	DALYs (Disability-Adjusted Life Years)	135	Brazil	3	Both	22	All ages	468	Testicular cancer	1	Number	2019	23191.507006918808	25914.223345788185	21516.97544236407
**2**	DALYs (Disability-Adjusted Life Years)	135	Brazil	3	Both	22	All ages	471	Kidney cancer	1	Number	2019	124141.0590143296	130542.36951187854	117594.37285171935
**2**	DALYs (Disability-Adjusted Life Years)	135	Brazil	3	Both	22	All ages	474	Bladder cancer	1	Number	2019	107756.97261264105	114563.36393598215	99750.28057181
**2**	DALYs (Disability-Adjusted Life Years)	135	Brazil	3	Both	22	All ages	477	Brain and central nervous system cancer	1	Number	2019	373186.94954039867	423714.96231683216	250314.8613896384
**2**	DALYs (Disability-Adjusted Life Years)	135	Brazil	3	Both	22	All ages	480	Thyroid cancer	1	Number	2019	30147.413408880282	34681.44923948814	28235.523143822324
**2**	DALYs (Disability-Adjusted Life Years)	135	Brazil	3	Both	22	All ages	483	Mesothelioma	1	Number	2019	27150.575510782634	31520.59250258649	23145.246818044227
**2**	DALYs (Disability-Adjusted Life Years)	135	Brazil	3	Both	22	All ages	484	Hodgkin lymphoma	1	Number	2019	26688.226578781345	33171.09543186858	23365.670642980742
**2**	DALYs (Disability-Adjusted Life Years)	135	Brazil	3	Both	22	All ages	485	Non-Hodgkin lymphoma	1	Number	2019	184737.13899070618	194056.62553230577	175931.06567941696
**2**	DALYs (Disability-Adjusted Life Years)	135	Brazil	3	Both	22	All ages	486	Multiple myeloma	1	Number	2019	95394.46113104426	104213.83006559599	83617.5144875994
**2**	DALYs (Disability-Adjusted Life Years)	135	Brazil	3	Both	22	All ages	487	Leukemia	1	Number	2019	312188.1218419523	330198.8875577887	294027.89333270973
**2**	DALYs (Disability-Adjusted Life Years)	135	Brazil	3	Both	22	All ages	490	Other neoplasms	1	Number	2019	32886.09407996156	37700.022170040975	23510.37606694146
**2**	DALYs (Disability-Adjusted Life Years)	135	Brazil	3	Both	22	All ages	492	Rheumatic heart disease	1	Number	2019	184224.8105900875	238145.94133204297	143687.28083481264
**2**	DALYs (Disability-Adjusted Life Years)	135	Brazil	3	Both	22	All ages	567	Depressive disorders	1	Number	2019	1606200.1060032342	2183261.418197973	1128433.2339183583
**2**	DALYs (Disability-Adjusted Life Years)	135	Brazil	3	Both	22	All ages	570	Bipolar disorder	1	Number	2019	560746.6417763463	863274.5302939996	344727.9253954897
**2**	DALYs (Disability-Adjusted Life Years)	135	Brazil	3	Both	22	All ages	571	Anxiety disorders	1	Number	2019	1640282.276806073	2235690.03439169	1140302.6663526746

**2**	DALYs (Disability-Adjusted Life Years)	135	Brazil	3	Both	22	All ages	572	Eating disorders	1	Number	2019	111577.63624441753	166250.93991236165	70643.35105135657
**2**	DALYs (Disability-Adjusted Life Years)	135	Brazil	3	Both	22	All ages	575	Autism spectrum disorders	1	Number	2019	113726.96609472959	164758.9937986317	74291.22683672752
**2**	DALYs (Disability-Adjusted Life Years)	135	Brazil	3	Both	22	All ages	578	Attention-deficit/hyperactivity disorder	1	Number	2019	47953.07915168324	83607.90952347431	26961.62766951483
**2**	DALYs (Disability-Adjusted Life Years)	135	Brazil	3	Both	22	All ages	579	Conduct disorder	1	Number	2019	128397.79235378384	204189.6598921387	71282.73772179394
**2**	DALYs (Disability-Adjusted Life Years)	135	Brazil	3	Both	22	All ages	582	Idiopathic developmental intellectual disability	1	Number	2019	34649.15127495471	62322.929554071205	12433.799834474295
**2**	DALYs (Disability-Adjusted Life Years)	135	Brazil	3	Both	22	All ages	585	Other mental disorders	1	Number	2019	241992.75685516954	367917.2957925287	155387.44700703857
**2**	DALYs (Disability-Adjusted Life Years)	135	Brazil	3	Both	22	All ages	587	Diabetes mellitus	1	Number	2019	2564212.667497648	2996547.819842529	2173111.0039907345
**2**	DALYs (Disability-Adjusted Life Years)	135	Brazil	3	Both	22	All ages	588	Acute glomerulonephritis	1	Number	2019	2404.2123428903255	2649.1618913404873	2176.540270872074
**2**	DALYs (Disability-Adjusted Life Years)	135	Brazil	3	Both	22	All ages	589	Chronic kidney disease	1	Number	2019	1184118.1273818228	1274555.0568420228	1093951.4233026316
**2**	DALYs (Disability-Adjusted Life Years)	135	Brazil	3	Both	22	All ages	674	Age-related and other hearing loss	1	Number	2019	1095613.7821147854	1585939.3202882975	720912.4810932735
**2**	DALYs (Disability-Adjusted Life Years)	135	Brazil	3	Both	22	All ages	679	Other sense organ diseases	1	Number	2019	99656.20970095783	153713.8384571698	59350.81853975275
**2**	DALYs (Disability-Adjusted Life Years)	135	Brazil	3	Both	22	All ages	680	Oral disorders	1	Number	2019	972705.4267380488	1481505.7294827788	600806.2060890334
**2**	DALYs (Disability-Adjusted Life Years)	135	Brazil	3	Both	22	All ages	686	Sudden infant death syndrome	1	Number	2019	21391.476831710075	27415.072183315606	16461.313607019594
**2**	DALYs (Disability-Adjusted Life Years)	135	Brazil	3	Both	22	All ages	689	Road injuries	1	Number	2019	2431395.629322869	2553712.411408228	2297711.291956654
**2**	DALYs (Disability-Adjusted Life Years)	135	Brazil	3	Both	22	All ages	958	Typhoid and paratyphoid	1	Number	2019	365.1909649474692	656.6235135431874	191.80890007258702
**2**	DALYs (Disability-Adjusted Life Years)	135	Brazil	3	Both	22	All ages	959	Invasive Non-typhoidal Salmonella (iNTS)	1	Number	2019	2642.4976643387245	4122.949522841808	1530.0588290879693
**2**	DALYs (Disability-Adjusted Life Years)	135	Brazil	3	Both	22	All ages	972	Headache disorders	1	Number	2019	1649717.1590034852	3747117.047268994	256902.55292963076
