# Supplementary material for: Disability-adjusted life years associated with COVID-19 in Brazil, 2020
Source: PLoS One. 2025 Mar 27;20(3):e0319941. doi: 10.1371/journal.pone.0319941 (PMC11949356; doi:10.1371/journal.pone.0319941)
Supplement: S2 Table — (PDF) [file pone.0319941.s002.pdf]

S2 Table. Morbidity sensitivity analysis: impact on COVID-19 YLD, Brazil, 2020

Impacted health states	Sensitivity	Total YLD
Mild to moderate cases	Decrease moderate cases and the duration by 50%	2,474.78
	Double moderate cases and the duration	39,596.60
Severe cases	Decrease severe cases and the duration by 50%	3,014.47
	Double severe cases and the duration	48,231.56
Critical cases	Decrease critical cases and the duration by 50%	2,699.87
	Double critical cases and the duration	43,197.98
Long covid	Decrease long covid and the duration by 50%	3,957.50
	Double long covid and the duration	63,320.09
**Combination of scenarios that minimise YLD impact [a]**		**12,146.64**
**Combination of scenarios that maximise YLD impact [b]**		**194,346.20**

a Combined criteria used: decrease in the duration and number of mild to moderate, severe, critical and the long covid cases by 50%;
b Combined criteria used: double the duration and the number of mild to moderate, severe, critical, and the long covid cases.
