# Supplementary material for: Disability-adjusted life years associated with COVID-19 in Brazil, 2020
Source: PLoS One. 2025 Mar 27;20(3):e0319941. doi: 10.1371/journal.pone.0319941 (PMC11949356; doi:10.1371/journal.pone.0319941)
Supplement: S3 Table — (PDF) [file pone.0319941.s003.pdf]

S3 Table. Total number of COVID-19 mild, severe and critical cases, long covid, Dalys and Dalys/100,000 person in Brazil by state between February 26, 2020, to December 31, 2020

Code_state	Mild	Severe	Critical	Long	Deaths	Daly	Daly100k
**11**	142,1	36,1	33,3	227,3	53451,1	53890,0	3128,1
**12**	56,2	12,8	0,2	89,8	21869,5	22028,6	2550,6
**13**	252,8	194,0	70,3	404,2	149009,7	149930,9	3638,0
**14**	30,6	19,9	4,5	48,9	25415,6	25519,4	3981,3
**15**	247,5	250,8	66,7	395,7	219675,9	220636,7	2606,4
**16**	79,8	22,5	11,1	127,7	32010,2	32251,3	4105,7
**17**	104,2	42,7	8,9	166,7	32191,6	32514,2	2114,2
**21**	118,8	73,5	30,1	189,9	124506,0	124918,2	1792,0
**22**	150,7	106,8	37,8	241,0	66192,8	66729,2	2000,4
**23**	342,6	292,3	95,5	547,9	280471,8	281750,1	3099,9
**24**	31,2	76,0	36,9	49,8	77178,6	77372,5	2270,6
**25**	351,5	119,7	53,6	562,2	85822,7	86909,8	2138,0
**26**	322,8	231,8	34,4	516,3	296086,7	297192,0	3148,6
**27**	195,1	55,4	54,4	312,0	92233,1	92850,0	2888,2
**28**	104,7	65,2	20,3	167,5	56809,4	57167,3	2536,0
**29**	792,9	231,8	152,3	1267,9	275203,2	277648,2	1882,5
**31**	838,8	878,1	270,2	1341,3	328761,2	332089,5	1579,0
**32**	197,7	44,0	62,6	316,1	118960,0	119580,4	2988,4
**33**	410,7	642,8	164,4	656,7	773862,2	775736,8	4504,3
**35**	2893,3	2704,3	1068,3	4626,8	1274248,9	1285541,7	2823,9
**41**	70,2	594,0	380,4	112,3	225039,5	226196,5	1956,5
**42**	976,9	259,5	98,8	1562,2	126360,9	129258,3	1713,9
**43**	909,0	447,4	322,9	1453,6	205281,5	208414,4	1857,5
**50**	193,9	159,0	30,4	310,1	54302,3	54995,8	1959,3
**51**	69,8	105,6	47,9	111,6	123885,9	124220,7	3439,1
**52**	680,6	215,5	148,8	1088,3	202726,7	204859,9	2901,6
**53**	164,6	233,0	132,5	263,2	86946,8	87740,2	3003,3
