# Supplementary material for: Disability-adjusted life years associated with COVID-19 in Brazil, 2020
Source: PLoS One. 2025 Mar 27;20(3):e0319941. doi: 10.1371/journal.pone.0319941 (PMC11949356; doi:10.1371/journal.pone.0319941)
Supplement: S1 Text — (PDF) [file pone.0319941.s004.pdf]

S1 Text. Code used in the analysis of the manuscript "Disability-adjusted life years associated with COVID-19 in Brazil, 2020"

```
# Severe cases COVID-19 ---------------------------------------------------
--

sari <- fread("/Users/cleber/2020", sep = ";", select = c("DT_SIN_PRI", "
CS_SEXO","SG_UF_NOT", "UTI","DT_INTERNA", "DT_ENCERRA", "NU_IDADE_N", "DT
_ENTUTI" , "DT_SAIDUTI")) #lendo os csv e sele

as.Date("01/01/2021", "%d/%m/%Y") - as.Date("25/02/2020", "%d/%m/%Y")

sari <- sari %>%
  mutate(duration_hosp = as.numeric(difftime(as.Date(DT_ENCERRA, "%d/%m/%
Y"), as.Date(DT_INTERNA, "%d/%m/%Y"), units = "days")),
         duration_hosp = replace(duration_hosp, duration_hosp > 311, 311)
,
         duration_ICU = as.numeric(difftime(as.Date(DT_SAIDUTI, "%d/%m/%Y
"), as.Date(DT_ENTUTI, "%d/%m/%Y"), units = "days")),
         duration_ICU = replace(duration_ICU, duration_ICU > 311, 311),
         age_group = case_when(NU_IDADE_N >=95 ~'95+ yo',
                               NU_IDADE_N >89 & NU_IDADE_N <95 ~'90-94yo'
,
                               NU_IDADE_N >84 & NU_IDADE_N <90 ~'85-89yo'
,
                               NU_IDADE_N >79 & NU_IDADE_N <85 ~'80-84yo'
,
                               NU_IDADE_N >74 & NU_IDADE_N <80 ~'75-79yo'
,
                               NU_IDADE_N >69 & NU_IDADE_N <75 ~'70-74yo'
,
                               NU_IDADE_N >64 & NU_IDADE_N <70 ~'65-69yo'
,
                               NU_IDADE_N >59 & NU_IDADE_N <65 ~'60-64yo'
,
                               NU_IDADE_N >54 & NU_IDADE_N <60 ~'55-59yo'
,
                               NU_IDADE_N >49 & NU_IDADE_N <55 ~'50-54yo'
,
                               NU_IDADE_N >44 & NU_IDADE_N <50 ~'45-49yo'
,
                               NU_IDADE_N >39 & NU_IDADE_N <45 ~'40-44yo'
,
                               NU_IDADE_N >34 & NU_IDADE_N <40 ~'35-39yo'
,
                               NU_IDADE_N >29 & NU_IDADE_N <35 ~'30-34yo'
,
                               NU_IDADE_N >24 & NU_IDADE_N <30 ~'25-29yo'
```

```
,
                              NU_IDADE_N >19 & NU_IDADE_N <25 ~'20-24yo'
,
                              NU_IDADE_N >14 & NU_IDADE_N <20 ~'15-19yo'
,
                              NU_IDADE_N >9 & NU_IDADE_N <15 ~'10-14yo',
                              NU_IDADE_N >4 & NU_IDADE_N <10 ~'5-9yo',
                              NU_IDADE_N >=1 & NU_IDADE_N <5 ~'1-4yo',
                              NU_IDADE_N < 1 ~'<1yo'),
         count=1) %>%
  filter(as.Date(DT_INTERNA, "%d/%m/%Y") > as.Date("25/02/2020", "%d/%m/%
Y") & as.Date(DT_INTERNA, "%d/%m/%Y") < as.Date("01/01/2021", "%d/%m/%Y")
,
         CS_SEXO=="F" | CS_SEXO=="M",
         NU_IDADE_N >= 0 & NU_IDADE_N < 120) %>%
  rename(Sex=CS_SEXO)

#mean time of hospitalization of non-ICU patients
sari %>%
  filter(UTI!=1,
         DT_INTERNA != "" & DT_ENCERRA != "",
         duration_hosp > 0) %>%
  summarise(mean(duration_hosp))

severe_covid_grouped <- sari %>%
  filter(UTI!=1,
         duration_hosp > 0) %>%
  group_by( Sex, age_group) %>%
  summarise(severe_cases = sum(count)) %>%
  mutate(Duration = 37.43 / 365, # the mean time of hospitalization in pe
rson-year
         DW = 0.133,
         severe_YLD = severe_cases * Duration* DW)

## `summarise()` has grouped output by 'Sex'. You can override using the
`.groups`
## argument.

severe_covid_grouped <- severe_covid_grouped %>%
  mutate(Sex=case_when(Sex=="F" ~ "Female",
                       Sex=="M" ~ "Male"))


# Critical (UCI COVID-19) ---------------------------------------------------
-------------

#mean time hospitalization UCI:
sari %>%
  filter(UTI==1,
```

```
         duration_ICU > 0) %>%
  summarise(mean(duration_ICU))

##   mean(duration_ICU)
## 1           11.16314

#grouping...
critical_covid_grouped <- sari %>%
  filter(UTI==1,
         duration_ICU > 0) %>% #filtering the cases transferred to UCI
  group_by(Sex, age_group) %>%
  summarise( uci_cases = sum(count)) %>%
  mutate(Duration = 11.16 / 365, # the mean time of hospitalization in pe
rson-year
         DW = 0.655,
         critical_YLD = uci_cases * Duration* DW)

## `summarise()` has grouped output by 'Sex'. You can override using the
`.groups`
## argument.

critical_covid_grouped <- critical_covid_grouped %>%
  mutate(Sex=case_when(Sex=="F" ~ "Female",
                       Sex=="M" ~ "Male"))
# Mild cases COVID-19 ---------------------------------------------------
--

library(foreach)

#Get sheets
setwd("/Users/cleber/2020")

mild_covid <- flu_like %>%
  filter(dataInicioSintomas > as.Date("2020-02-25") & dataInicioSintomas
< as.Date("2021-01-01"), #filtering the cases with symptom between the fi
rst cases of COVID-19 notified in BR and the end of 2020
         sexo=="Feminino" | sexo=="Masculino", #excluding the records wit
hout gender classification
         idade >= 0 & idade < 120,  #excluding the records with age with
negative values or >120yo
         complete.cases(idade)) %>%
  mutate(classif = case_when(
    str_detect(classificacaoFinal,
               pattern = "Confir") ~ "Confirmed",
    classificacaoFinal== "Descartado" ~"Discarted",
    classificacaoFinal== "Suspeito" ~ "Suspect",
    classificacaoFinal== "" ~ "NA",
    TRUE~"Other"
  )) %>%
  filter(classif == "Confirmed" | classif == "Other") %>% #excluding the
```

```
non-COVID records
  mutate(
    age_group = case_when(idade >=95 ~'95+ yo', #creating age-groups
                          idade >89 & idade <95 ~'90-94yo',
                          idade >84 & idade <90 ~'85-89yo',
                          idade >79 & idade <85 ~'80-84yo',
                          idade >74 & idade <80 ~'75-79yo',
                          idade >69 & idade <75 ~'70-74yo',
                          idade >64 & idade <70 ~'65-69yo',
                          idade >59 & idade <65 ~'60-64yo',
                          idade >54 & idade <60 ~'55-59yo',
                          idade >49 & idade <55 ~'50-54yo',
                          idade >44 & idade <50 ~'45-49yo',
                          idade >39 & idade <45 ~'40-44yo',
                          idade >34 & idade <40 ~'35-39yo',
                          idade >29 & idade <35 ~'30-34yo',
                          idade >24 & idade <30 ~'25-29yo',
                          idade >19 & idade <25 ~'20-24yo',
                          idade >14 & idade <20 ~'15-19yo',
                          idade >9 & idade <15 ~'10-14yo',
                          idade >4 & idade <10 ~'5-9yo',
                          idade >=1 & idade <5 ~'1-4yo',
                          idade < 1 ~'<1yo'))

mild_covid_grouped <- mild_covid %>%
  mutate(count=1) %>%
  group_by(sexo, age_group) %>%  #grouping by sex, CS_SEXO, age_group and
age
  rename(Sex = sexo) %>%
  summarise(symp_cases = sum(count)) %>%
  mutate(Sex=case_when(Sex=="Feminino" ~ "Female",
                       Sex=="Masculino" ~ "Male"))#%>%

severe_covid_grouped <- severe_covid_grouped %>%
  mutate(Sex=case_when(Sex=="F" ~ "Female",
                       Sex=="M" ~ "Male"))


mild_correct <- mild_covid_grouped %>% right_join(severe_covid_grouped)#m
erge(severe_merging, mild, by=c("Sex", "age_group"))

## Joining, by = c("Sex", "age_group")

mild_correct <- mild_correct %>%
  mutate(correct_mild = symp_cases - severe_cases)

mild <- mild_correct %>%
  select(Sex, age_group,correct_mild) %>%
  mutate(Duration = 10/365, #10 days divided into 365 (i.e., person-year)
         DW = 0.051,
```

```
        mild_YLD = correct_mild* Duration* DW) %>%
  select(Sex, age_group, correct_mild, mild_YLD) %>%
  rename(mild_cases = correct_mild)

# Long COVID -----------------------------------------------------------------
--



long <- mild_correct %>%
  select(Sex, age_group, correct_mild) %>%
  mutate(long_cases = correct_mild*0.133,
         Duration = 28/365,
         DW = 0.219,
         long_YLD = long_cases* Duration* DW)  %>%
  select(Sex, age_group,long_cases, long_YLD)




# Deaths due to COVID-19 -----------------------------------------------------
--

mortality<- fread("/Users/cleber/2020", sep=";", select = c("CAUSABAS", "
IDADE", "SEXO", "CODMUNRES"))

mortality_covid <- mortality %>%
  filter(complete.cases(IDADE) & (SEXO==1 | SEXO==2)) %>%
  filter(str_detect(CAUSABAS, pattern = "U071")|str_detect(CAUSABAS, patt
ern = "U072")|
           str_detect(CAUSABAS, pattern = "U109")|str_detect(CAUSABAS, pa
ttern = "B342")| str_detect(CAUSABAS, pattern = "B972")%>%
  mutate(UF = substr(CODMUNRES, 1, 2),
         UF =  case_when(UF == 11 ~"RO",
                         UF == 12 ~"AC",
                         UF == 13 ~"AM",
                         UF == 14 ~"RR",
                         UF == 15 ~"PA",
                         UF == 16 ~"AP",
                         UF == 17 ~"TO",
                         UF == 21 ~"MA",
                         UF == 22 ~"PI",
                         UF == 23 ~"CE",
                         UF == 24 ~"RN",
                         UF == 25 ~"PB",
                         UF == 26 ~"PE",
                         UF == 27 ~"AL",
                         UF == 28 ~"SE",
                         UF == 29 ~"BA",
                         UF == 31 ~"MG",
                         UF == 32 ~"ES",
```

```
                         UF == 33 ~"RJ",
                         UF == 35 ~"SP",
                         UF == 41 ~"PR",
                         UF == 42 ~"SC",
                         UF == 43 ~"RS",
                         UF == 50 ~"MS",
                         UF == 51 ~"MT",
                         UF == 52 ~"GO",
                         UF == 53 ~"DF"),
         age_group = case_when(IDADE < 401 ~'0-1yo',
                               IDADE >=495 ~'95+ yo',
                               IDADE >=490 & IDADE <=494 ~'90-94yo',
                               IDADE >=485 & IDADE <=489 ~'85-89yo',
                               IDADE >=480 & IDADE <=484 ~'80-84yo',
                               IDADE >=475 & IDADE <=479 ~'75-79yo',
                               IDADE >=470 & IDADE <=474 ~'70-74yo',
                               IDADE >=465 & IDADE <=469 ~'65-69yo',
                               IDADE >=460 & IDADE <=465 ~'60-64yo',
                               IDADE >=455 & IDADE <=459 ~'55-59yo',
                               IDADE >=450 & IDADE <=454 ~'50-54yo',
                               IDADE >=445 & IDADE <=449 ~'45-49yo',
                               IDADE >=440 & IDADE <=444 ~'40-44yo',
                               IDADE >=435 & IDADE <=439 ~'35-39yo',
                               IDADE >=430 & IDADE <=434 ~'30-34yo',
                               IDADE >=425 & IDADE <=429 ~'25-29yo',
                               IDADE >=420 & IDADE <=424 ~'20-24yo',
                               IDADE >=415 & IDADE <=419 ~'15-19yo',
                               IDADE >=410 & IDADE <=414 ~'10-14yo',
                               IDADE >=405 & IDADE <=409 ~'5-9yo',
                               IDADE >=401 & IDADE <=404 ~'1-4yo'))
rm(mortality)
yll <- mortality_covid %>%
  mutate(count=1) %>%
  group_by(age_group,SEXO) %>%
  summarise(deaths = sum(count)) %>%
  mutate(GBD_lifetable = case_when(
    age_group =='0-1yo' ~ 88.8718951,
    age_group =='1-4yo' ~ 88.00051053,
    age_group =='5-9yo' ~ 84.03008056,
    age_group =='10-14yo' ~ 79.04633476,
    age_group =='15-19yo' ~ 74.0665492,
    age_group =='20-24yo' ~ 69.10756792,
    age_group =='25-29yo' ~ 64.14930031,
    age_group =='30-34yo' ~ 59.1962771,
    age_group =='35-39yo' ~ 54.25261364,
    age_group =='40-44yo' ~ 49.31739311,
    age_group =='45-49yo' ~ 44.43332057,
    age_group =='50-54yo' ~ 39.63473787,
    age_group =='55-59yo' ~ 34.91488095,
    age_group =='60-64yo' ~ 30.25343822,
```

```r
    age_group =='65-69yo' ~ 25.68089534,
    age_group =='70-74yo' ~ 21.28820012,
    age_group =='75-79yo' ~ 17.10351469,
    age_group =='80-84yo' ~ 13.23872477,
    age_group =='85-89yo' ~ 9.990181244,
    age_group =='90-94yo' ~ 7.617724915,
    age_group =='95+ yo' ~ 5.922359078),
    yll=deaths*GBD_lifetable
  )



# Mergin the results ----------------------------------------------------
--

deaths <- yll %>%
  mutate(Sex=case_when(SEXO==2 ~ "Female",
                       SEXO==1 ~ "Male")) %>%
  select(age_group, Sex, deaths, yll)

severe <- severe_covid_grouped %>%
  select(age_group,Sex,severe_cases, severe_YLD)

critical <- critical_covid_grouped %>%
  select(age_group, Sex, uci_cases, critical_YLD) %>%
  rename(critical_cases = uci_cases)


daly1 <- merge(critical, severe, by=c("Sex", "age_group"))

daly <- mild %>% right_join(daly1)

## Joining, by = c("Sex", "age_group")

daly <- long %>% right_join(daly)

## Joining, by = c("Sex", "age_group")

daly <- deaths %>% right_join(daly)

## Joining, by = c("age_group", "Sex")

daly[is.na(daly)]<-0

daly <- daly %>%
  mutate(Daly = mild_YLD+severe_YLD+critical_YLD+long_YLD+yll)
```

```r
# Calculating uncertainty intervals ----------------------------------------------
------------


mild_covid_ <- mild_covid %>%
  select(age_group, sexo)

Incidence_mild <- table(mild_covid_$age_group, mild_covid_$sexo)
Incidence_mild <-as.data.frame(Incidence_mild)

Incidence_mild_female <- Incidence_mild %>% filter( Incidence_mild$Var2 =
= "Feminino")
Incidence_mild_male <- Incidence_mild %>% filter( Incidence_mild$Var2 ==
"Masculino")

Incidence_mild <- rbind.data.frame(Incidence_mild_female,Incidence_mild_m
ale)

Duration = 10 / 365

DW = 0.051

mean_median_ci <- function(x) {
  c(mean = mean(x),
    median = median(x),
    quantile(x, probs = c(0.025, 0.975)))
}

YLDmildUncertainty <- foreach(i = 1:nrow(Incidence_mild),.combine = rbind
) %do% {
  Mean <- Incidence_mild[i,3]
  DW <- sample(x = rpert(n= 10000, x.min= 0.032, x.mode= 0.051, x.max= 0.
074), size = 10000,replace = TRUE)
  mean_median_ci(Mean * DW * Duration) }

colSums(YLDmildUncertainty)

##      mean    median      2.5%     97.5%
## 10871.375 10837.277  7851.139 14076.304

YLDmildUncertainty <- as.data.frame(YLDmildUncertainty)


long_covid <- mild_covid %>%
  select(age_group, sexo)

Incidence_long <- table(long_covid$age_group, long_covid$sexo)
Incidence_long <-as.data.frame(Incidence_long)
```

```r
Incidence_long$long_cases <- Incidence_long$Freq*0.133

Incidence_long_female <- Incidence_long %>% filter( Incidence_long$Var2 =
= "Feminino")
Incidence_long_male <- Incidence_long %>% filter( Incidence_long$Var2 ==
"Masculino")

Incidence_long <- rbind.data.frame(Incidence_long_female,Incidence_long_m
ale)


Duration = 28 / 365

DW = 0.219
mean_median_ci <- function(x) {
  c(mean = mean(x),
    median = median(x),
    quantile(x, probs = c(0.025, 0.975)))
}

YLDlongUncertainty <- foreach(i = 1:nrow(Incidence_long),.combine = rbind
) %do% {
  Mean <- Incidence_long[i,4]
  DW <- sample(x = rpert(n= 10000, x.min= 0.148, x.mode= 0.219, x.max= 0.
308), size = 10000,replace = TRUE)
  mean_median_ci(Mean * DW * Duration) }

colSums(YLDlongUncertainty)

##     mean   median     2.5%    97.5%
## 17394.29 17344.28 13108.60 21966.77

YLDlongUncertainty <- as.data.frame(YLDlongUncertainty)


sari_ <- sari %>%
  filter(UTI!=1,
         DT_INTERNA != "" & DT_ENCERRA != "",
         duration_hosp > 0) %>%
  select(age_group, Sex, count)

Incidence_hosp <- table(sari_$age_group, sari_$Sex,sari_$count)
Incidence_hosp <-as.data.frame(Incidence_hosp)

Incidence_hosp_female <- Incidence_hosp %>% filter( Incidence_hosp$Var2 =
= "F")
Incidence_hosp_male <- Incidence_hosp %>% filter( Incidence_hosp$Var2 ==
"M")
```

```
Incidence_hosp2 <- merge(Incidence_hosp_female,Incidence_hosp_male,by="Va
r1")
Incidence_hosp3 <- rbind.data.frame(Incidence_hosp_female,Incidence_hosp_
male)

Duration = 37.43 / 365

DW = 0.133

mean_median_ci <- function(x) {
  c(mean = mean(x),
    median = median(x),
    quantile(x, probs = c(0.025, 0.975)))
}

YLDhospUncertainty <- foreach(i = 1:nrow(Incidence_hosp3),.combine = rbin
d) %do% {
  Mean1 <- Incidence_hosp3[i,4]
  DW <- sample(x = rpert(n= 10000, x.min= 0.088, x.mode= 0.133, x.max= 0.
190), size = 10000,replace = TRUE)
  mean_median_ci(Mean1 * DW * Duration) }

colSums(YLDhospUncertainty)

##      mean    median      2.5%     97.5%
##  8236.606  8204.707  6124.039 10501.927

YLDhospUncertainty <- as.data.frame(YLDhospUncertainty)

# Sensitivity -----------------------------------------------------------
--

mild_sens_double <- mild_correct %>%
  select(Sex, age_group,correct_mild) %>%
  mutate(Duration = 10/365, #10 days divided into 365 (i.e., person-year)
         DW = 0.051,
         mild = (correct_mild*2)* (Duration*2)* DW) %>%
  select(Sex, age_group,mild)
sum(mild_sens_double_cases$mild)

#39596.6


long_sens_double <- mild_correct %>%
  select(Sex, age_group, correct_mild) %>%
  mutate(long_cases = (correct_mild*2)*0.133,
         Duration = 28/365,
         DW = 0.219,
         long = long_cases* (Duration*2)* DW)  %>%
```

```
  select(Sex, age_group,long)
sum(long_sens_double_cases$long)

#63320.09


severe_sens_double <- severe %>%
  summarise(severe_cases=sum(severe)) %>%
  mutate(Duration = as.numeric(severe_cases) /365,
         DW = 0.133,
         severe = (severe_cases*2)* (Duration*2)* DW)
sum(severe_sens_double$severe)

# 48231.56

critical_sens_double <- critical %>%
  summarise(critical_cases=sum(critical)) %>%
  mutate(Duration = as.numeric(critical_cases) /365,
         DW = 0.655,
         critical = (critical_cases*2)* (Duration*2)* DW)
sum(critical_sens_double$critical)

# 43197.98



mild_sens_half <- mild_correct %>%
  select(Sex, age_group,correct_mild) %>%
  mutate(Duration = 10/365, #10 days divided into 365 (i.e., person-year)
         DW = 0.051,
         mild = (correct_mild/2)* (Duration/2)* DW) %>%
  select(Sex, age_group,mild)
sum(mild_sens_half_cases$mild)

#2474.788

long_sens_half <- mild_correct %>%
  select(Sex, age_group, correct_mild) %>%
  mutate(long_cases = (correct_mild/2)*0.133,
         Duration = 28/365,
         DW = 0.219,
         long = long_cases* (Duration/2)* DW)  %>%
  select(Sex, age_group,long)
sum(long_sens_half_cases$long)

#3957.506

severe_sens_half <- severe %>%
  summarise(severe_cases=sum(severe)) %>%
  mutate(Duration = as.numeric(severe_cases) /365,
         DW = 0.133,
```

```
         severe = (severe_cases/2)* (Duration/2)* DW)
sum(severe_sens_half$severe)

# 3014.473

critical_sens_half <- critical %>%
  summarise(critical_cases=sum(critical)) %>%
  mutate(Duration = as.numeric(critical_cases) /365,
         DW = 0.655,
         critical = (critical_cases/2)* (Duration/2)* DW)
sum(critical_sens_half$critical)

# 2699.874


sari_ <- sari %>%
  filter(UTI==1,
         duration_ICU > 0) %>%
  select(age_group, Sex, UTI)

Incidence_ICU <- table(sari_$age_group, sari_$Sex)
Incidence_ICU <-as.data.frame(Incidence_ICU)

Incidence_ICU_female <- Incidence_ICU %>% filter( Incidence_ICU$Var2 == "
F")
Incidence_ICU_male <- Incidence_ICU %>% filter( Incidence_ICU$Var2 == "M"
)

Incidence_ICU <- rbind.data.frame(Incidence_ICU_female,Incidence_ICU_male
)

critical_covid_grouped <- sari %>%
  filter(UTI==1,
         duration_ICU > 0) %>% #filtering the cases transferred to UCI
  group_by(Sex, age_group) %>%
  summarise( uci_cases = sum(count))

## `summarise()` has grouped output by 'Sex'. You can override using the
`.groups`
## argument.

Duration = 11.16 / 365

DW = 0.655

mean_median_ci <- function(x) {
  c(mean = mean(x),
    median = median(x),
    quantile(x, probs = c(0.025, 0.975)))
}
```

```
YLDICUUncertainty <- foreach(i = 1:nrow(Incidence_ICU),.combine = rbind)
%do% {
  Mean <- Incidence_ICU[i,3]
  DW <- sample(x = rpert(n= 10000, x.min= 0.579, x.mode= 0.655, x.max= 0.
727), size = 10000,replace = TRUE)
  mean_median_ci(Mean * DW * Duration) }

colSums(YLDICUUncertainty)

##     mean   median     2.5%    97.5%
## 3433.935 3434.875 3156.430 3706.263

YLDICUUncertainty <- as.data.frame(YLDICUUncertainty)
```
